# Milk Alkali and Hydrochlorothiazide: A Case Report

**DOI:** 10.1155/2011/729862

**Published:** 2011-05-23

**Authors:** Babar Parvez, Chinenye Emuwa, Marquetta L. Faulkner, John J. Murray

**Affiliations:** Department of Internal Medicine, Meharry Medical College, 1818 Albion Street, Nashville, TN 37208, USA

## Abstract

Hypercalcemia is a relatively common clinical problem in both outpatient and inpatient settings. Primary pathophysiology is the entry of calcium that exceeds its excretion into urine or deposition in bone into circulation. Among a wide array of causes of hypercalcemia, hyperparathyroidism and malignancy are the most common, accounting for greater than 90 percent of cases. Concordantly, there has been a resurgence of milk-alkali syndrome associated with the ingestion of large amounts of calcium and absorbable alkali, making it the third leading cause of hypercalcemia (Beall and Scofield, 1995 and Picolos et al., 2005). This paper centers on a case of over-the-counter calcium and alkali ingestion for acid reflux leading to milk alkali with concordant use of thiazide diuretic for hypertension.

## 1. Introduction

Sippy, almost 100 years ago, developed a calcium-laden milk and antacid regimen for the treatment of peptic ulcer disease [1]. The rationale was to neutralize the hyperacidity that was deemed responsible for peptic ulcer disease. The Sippy regimen until the 1970s was used for the treatment of peptic ulcer disease, a disorder that was predominant in middle-aged men, when nonantacid treatment was first introduced [1, 2]. Sippy's original recommendation consisted of the hourly administration of milk and cream together with what became known as the Sippy powders, which were 10 grains (1 grain = 65 mg) each of heavily calcinated magnesia and sodium bicarbonate, alternating with a powder that contained 10 grains of bismuth subcarbonate and 20 to 30 grains of sodium bicarbonate [1].

Hardt and Rivers in 1923 gave a detailed description of the toxicity that was associated with the antacid and milk regimen [3]. The symptoms consisted of nausea, vomiting, headache, dizziness, musculoskeletal pains, and weakness followed by prostration. There was an associated elevation of blood urea nitrogen (BUN) and serum creatinine, alkalosis, and albuminuria. Cope in 1936 first recognized hypercalcemia as a major feature of this toxicity [4]. He also showed a rapid resolution of alkalosis and hypercalcemia after cessation of treatment, but the recovery of renal function was much slower. Fundamentally, the differentiation of milk-alkali syndrome from primary hyperparathyroidism was evident due to the absence of hypophosphatemia and the resolution of the hypercalcemia once treatment with antacids was stopped. Burnett et al. in 1949 described a chronic variant of the milk-alkali syndrome in which renal failure was persistent and soft tissue calcifications were extensive [5]. 

Punsar and Somer in 1963 classified milk-alkali syndrome into three different types: acute, subacute (Cope's syndrome), and chronic (Burnett's syndrome) toxicity [6]. Shortly thereafter, McMillan and Freeman formulated a comprehensive table and compared clinical presentation, biochemical findings, and resolution of the three different types of the milk-alkali syndrome [7]. The subacute form (Cope's syndrome) was seen during therapy with calcium and alkali that had been used intermittently for years. In contrast to the acute form, there was less rapid improvement in symptoms and the recovery of renal function was slower. The chronic form (Burnett's syndrome) occurred after a long history of ingestion of large amounts of calcium and alkali. Presentation of Burnett's syndrome included complains of pruritus, diffuse musculoskeletal symptoms, nephrocalcinosis, and band keratopathy with large soft tissue calcium deposits [5, 8]. Although symptoms usually improved with the discontinuation of calcium antacids, the resolution of hypercalcemia was slow, especially in patients with large soft tissue deposits of calcium. Renal insufficiency often improved but did not resolve completely.

## 2. Case Presentation

Our patient is a 64-year-old Caucasian male who presented to the emergency room (ER) with a 2-week complaint of progressive weakness, dizziness on ambulation, nausea, and per accompanying family members, a deterioration in mental status. In the ER, he was hemodynamically stable, but serum analysis was significant for hypercalcemia of 16.9 with corrected calcium per serum albumin 18.2 (normal 8.4–10.2 milligrams per deciliter (mg/dL), formula for corrected serum calcium = measured serum calcium + 0.8 ∗ (normal albumin – measured patient albumin)), alkalosis with bicarbonate 34.6 (normal 21.0–31.0 millimole per liter (mmol/L), renal insufficiency with BUN 30 (normal 6–20 mg/dL) and creatinine 4.0 (normal 0.5–1.2 mg/dL), total protein 5.8 (normal 6.2–8.2 g/dL), albumin 3.0 (normal 3.2–5.0 g/dL), phosphorus 3.1 (normal 2.5–4.6 mg/dL), WBC 11.5 (normal 4.5–10.0 K/cumm), hemoglobin 14.3 (normal 13.0–17.0 g/dL), hematocrit 40.9 (normal 39.0–52.0), and platelets 341 (normal 140–400 K/cumm). Electrocardiogram (ECG) showed sinus tachycardia at 106 beats per minute, normal PR, and QRS intervals with QT/QTc 304/400 msec. Arterial blood gas showed alkalemia: pH 7.48 (normal 7.35–7.45), pCO^2^ 46 (normal 35–45 mm/Hg), and O^2^ saturation 99% (normal 95–98). 

With this physical presentation, hypercalcemia with renal insufficiency and alkalosis, he was admitted to acute medicine floor. Management included aggressive fluid resuscitation with normal saline, acute coronary syndrome rule out, telemetry placement for cardiac arrhythmia monitoring, and workup for the etiology of hypercalcemia. On thorough history and chart review, the patient admitted to taking large quantities of over-the-counter calcium supplementation in the form of TUMS for his chronic acid reflux spanning over 3 decades. Recently, over the past several weeks, due to worsening reflux symptoms, he had increased his daily TUMS from 5-6 tablets to as much as 20–30 tablets per day. He had previously been seen in a cardiology clinic (6 months prior to this presentation) for followup of hypertension (HTN) and was started on low-dose hydrochlorothiazide 12.5 mg per day. He was seen in a pulmonary clinic for followup of lung nodule (which had previously been worked up as benign) and in a gastrointestinal clinic 2 months after cardiology visit and was noted to have serum Ca of 11.6. He was counseled extensively on stopping over the counter acid suppression and given prescription of omeprazole 40 mg BID which he decided not to take. 

Etiology workup revealed ionized calcium (iCa) at 8.3 (normal 4.48–5.28 mg/dL), intact parathyroid hormone (iPTH) < 3.0 (normal 10–65 pg/mL) indicating non-PTH-mediated hypercalcemia, parathyroid-hormone-related peptide (PTHrp) at 0, Vit D 1,25 <8 (normal 18–64 pg/mL), and Vit D 25 at 23 (optimum level 25–80 ng/mL) ruling out malignancy and hypervitaminosis. Other causes were also ruled out with normal serum thyroid studies, serum protein electrophoresis, chest radiograph, and chest CT showing no new changes and renal ultrasound being benign. Gastroenterology and nephrology consults were obtained to conclude the diagnosis of milk-alkali syndrome and to formulate multidisciplinary plan. He underwent esophagogastroduodenoscopy (EGD) which showed grade IV reflux esophagitis, distal esophageal stricture, gastritis, and erosive duodenitis. Pathologic specimen was reported as benign squamous hyperplasia with negative stain for *H. pylori*. Initial fluid resuscitation was supplemented with IV proton pump inhibitor twice a day and nutrition evaluation and recommendation; all of which resulted in impressive resolution of hypercalcemia and restoration of renal functions to normal thus confirming milk-alkali syndrome. Patient was later discharged home on omeprazole 40 mg twice a day (BID) and hydrochlorothiazide 12.5 mg. As followup, he was seen in internal medicine clinic 3 months after discharge with serum Ca of 10.6, albumin 4.3, improvement in reflux symptoms, and baseline functional status. 

## 3. Discussion

In the milk-alkali syndrome, hypercalcemia develops because the input of calcium (dietary intake and intestinal absorption) exceeds the output (primarily renal excretion). About 4 to 60 g/d of calcium carbonate has been reported to induce the milk-alkali syndrome, indicating that factors besides calcium intake contribute to the development of the milk-alkali syndrome [9]. Factors that can increase calcium input include increased dietary calcium intake, ingestion of supplemental calcium, enhanced intestinal absorption of calcium usually resulting from stimulation by vitamin D that is present in some calcium supplements, and other dietary factors. Calcium-containing compounds have also been shown to directly increase gastric acid secretion by stimulating the CaSR (calcium-sensing receptor) when ingested orally, which in turn increases the availability of free calcium for absorption [10, 11].

It is likely that bone, the primary repository for calcium, increases calcium uptake but becomes saturated during high calcium exposure. The capacity of bone to increase calcium uptake per unit of bone changes with age as does the extent of bone remodeling [12, 13]. Randall et al. in 1961 gave insight into the time course of this syndrome after observing 40 men with peptic ulcer disease who were treated with milk and either aluminum hydroxide or calcium carbonate [8]. In the milk–carbonate group (12 grams of calcium per day), the mean plasma calcium concentration rose within 24 hours from 10 mg/dL to 11 mg/dL (2.5 to 2.75 mmol/L). In 9 of 20 patients treated for one week, the plasma calcium concentration reached or exceeded 11.3 mg/dL (2.8 mmol/L) and, in a few cases, exceeded 12 mg/dL (3 mmol/L). The mean plasma bicarbonate concentration rose slightly, but in three of the 20 patients exceeded 31.5 meq/L. The plasma creatinine concentration rose from 1.2 to 1.4 mg/dL (106 to 124 mmol/L) on the third day. The plasma phosphate concentration rose during the first few days. One patient developed the acute milk-alkali syndrome with hypercalcemia preceding the development of renal insufficiency and alkalosis. From this study, it can be concluded that although exposure to excessive calcium and alkali ingestion can cause marked increases in serum Ca and bicarbonate in rare cases, mild intermittent hypercalcemia can be seen in a significant number of patients. 

Renal vasoconstriction secondary to hypercalcemia leads to a fall in glomerular filtration rate, and hypercalcemia directly stimulates tubular hydrogen ion secretion and, therefore, bicarbonate reabsorption leading to metabolic alkalosis. The alkalosis helps to maintain hypercalcemia and volume depletion by enhancing the activity of the CaSR and by increasing calcium reabsorption in the distal tubule via the pH-sensitive calcium channel, the transient receptor potential vanilloid member 5 [TRPV5] [17–19]. Volume contraction due to vomiting, diuretics, or the natriuretic effects of hypercalcemia further aggravates hypercalcemia and alkalosis. Marked hypercalcemia can lead to the patient presenting with acute toxicity (nausea, vomiting, weakness, and mental changes with psychosis or depressed sensorium). 

After extensive clinical and laboratory evaluation, no evidence of malignant disease or primary hyperparathyroidism was seen in our patient. No cause of hypercalcemia could be identified other than the ingestion of large amount of CaCO_3_ (calcium carbonate) with concurrent use of thiazide diuretic. Along with the pathophysiology of hypercalcemia secondary to large calcium intake and absorption as mentioned above, thiazide diuretics are known to reduce urinary calcium excretion and produce mild hypercalcemia [16, 17]. In the kidney, distal convoluted tubule reabsorbs about 8% of the filtered Ca load. This occurs via epithelial Ca^2+^ channels. In the steady state, the cell must extrude all penetrating Ca, which occurs via a Ca^2+^ ATPase and through Na^+^/Ca^2+^ exchanger located on the basolateral surface of the cells of the distal tubule. Thiazides inhibit the Na^+^/Cl^−^ symport in the early distal convoluted tubule, thus causing a decrease in intracellular Na. This, in turn, enhances the activity of the Na^+^/Ca^2+^ exchanger, creating an increased driving for Ca resorption through the epithelial Ca^2+^ channels. The final effects increased Ca reabsorption leading to hypercalcemia. In a novel study, Nijenhuis et al. showed that mice that lacked active distal Ca^2+^ reabsorption (TRVP5-knockout) developed hypercalcemia with hydrochlorothiazide (HCTZ) treatment and that enhanced passive Ca^2+^ transport in the proximal tubule rather than active Ca^2+^ transport in distal convolution explains thiazide-induced hypocalciuria [20].

In conclusion, the ingestion of absorbable calcium, metabolic alkalosis, and concomitant use of thiazide diuretic can produce severe hypercalcemia which can be potentially more life threatening than is generally observed with either agent alone. This must be taken into account while prescribing thiazide diuretics for hypertension to patient with gastric reflux disease due to the ready availability of nonprescription medication containing CaCO_3_. 
